# A251 USE OF INDIRECT CALORIMETRY TESTING TO DIRECT NUTRITION SUPPORT IN CRITICALLY ILL PATIENTS WITH GASTROINTESTINAL CONDITIONS

**DOI:** 10.1093/jcag/gwab049.250

**Published:** 2022-02-21

**Authors:** L Russell, N Janisse, G Jones, T Karachi, P E Serrano, M ApSimon, D Armstrong, M I Pinto-Sanchez

**Affiliations:** 1 McMaster University, Hamilton, ON, Canada; 2 Hamilton Health Sciences, Hamilton, ON, Canada

## Abstract

**Background:**

Indirect calorimetry (IC), which measures oxygen uptake and carbon dioxide output, determines energy expenditure (EE) more precisely than predictive equations in critically ill patients. It is unknown whether the use of IC affects energy provision in critically ill patients with gastrointestinal (GI) conditions that affect absorption and digestion

**Aims:**

To (1) compare IC and predictive equations for determining energy needs and (2) evaluate whether IC results affect changes in nutrition support in critically ill patients with GI conditions.

**Methods:**

In a prospective, observational study, IC was performed for 25 to 55 mins in critically ill patients admitted to intensive care or clinical wards at 2 tertiary-care hospitals in Hamilton, Ontario between Feb 2018 to Sept 2021. EE measured by IC was compared to EE determined by a predictive equation (25 kcal/kg) or the Harris-Benedict (HB) formula. A change in energy provision was defined as a change of >10% directed by IC. Continuous data are expressed as means and standard deviation (SD), and categorical data as a proportion of patients. The Mann Whitney U Test (SPSSv26) was used to compare GI and non-GI populations.

**Results:**

Of 296 IC tests in 229 patients, 39 of them were in 30 GI patients (11 female; mean age 62 yrs; SD 19). Admission GI diagnoses were pancreatitis (33%), liver disease (20%), Crohn’s disease/ autoimmune enteropathy (20%), post-bowel resection (10%), chronic abdominal pain (10%), and cholangitis (7%). The predictive formula underestimated EE in 67% of GI patients (mean deficit 503 kcal/day) compared to IC, corresponding to a mean deficit of 25% of patients’ energy needs. The HB formula underestimated EE in 73% of patients (mean deficit 652 kcal/day), a mean deficit of 28% of patients’ energy needs compared to IC. Pancreatitis was the majority diagnosis (75% of the predictive equation; 50% HB) among patients with the highest deficit (>30%) in energy needs when compared to IC. There were no significant differences in the rates of underestimation of energy needs based on predictive and HB formulas between the GI and non-GI patients or between luminal GI and non-luminal GI conditions. After IC, 63% of tests led to changes in energy provisions in GI patients; most requiring an increase in energy provisions (53%).

**Conclusions:**

The use of IC to accurately measure EE led to changes in energy provisions in critically ill GI patients. Preventing over- and underfeeding with the implementation of IC to guide nutrition has the potential to improve outcomes in critically ill patients with gastrointestinal conditions.

**Funding Agencies:**

None

